# An 8-SNP LDL Cholesterol Polygenic Score: Associations with Cardiovascular Risk Traits, Familial Hypercholesterolemia Phenotype, and Premature Coronary Heart Disease in Central Romania

**DOI:** 10.3390/ijms251810038

**Published:** 2024-09-18

**Authors:** Ion Bogdan Mănescu, Manuela Rozalia Gabor, George Valeriu Moldovan, László Hadadi, Adina Huțanu, Claudia Bănescu, Minodora Dobreanu

**Affiliations:** 1 Department of Laboratory Medicine, Faculty of Medicine, George Emil Palade University of Medicine, Pharmacy, Science, and Technology of Targu Mures, 540142 Targu Mures, Romania; adina.hutanu@umfst.ro (A.H.); minodora.dobreanu@umfst.ro (M.D.); 2 Department of Economic Science, Faculty of Economics and Law, George Emil Palade University of Medicine, Pharmacy, Science, and Technology of Targu Mures, 540566 Targu Mures, Romania; manuela.gabor@umfst.ro; 3 Department of Economic Research, Centre for Law, Economics and Business Studies, George Emil Palade University of Medicine, Pharmacy, Science, and Technology of Targu Mures, 540566 Targu Mures, Romania; 4 Clinical Laboratory, Emergency County Clinical Hospital of Targu Mures, 540136 Targu Mures, Romania; 5 Emergency Institute for Cardiovascular Diseases and Transplantation, 540136 Targu Mures, Romania; hadadi.laszlo@ibcvt.ro; 6 Department of Genetics, Faculty of Medicine, George Emil Palade University of Medicine, Pharmacy, Science, and Technology of Targu Mures, 540142 Targu Mures, Romania; claudia.banescu@umfst.ro; 7 Genetics Laboratory, Center for Advanced Medical and Pharmaceutical Research, George Emil Palade University of Medicine, Pharmacy, Science, and Technology of Targu Mures, 540142 Targu Mures, Romania; 8 Immunology Laboratory, Center for Advanced Medical and Pharmaceutical Research, George Emil Palade University of Medicine, Pharmacy, Science, and Technology of Targu Mures, 540142 Targu Mures, Romania

**Keywords:** coronary heart disease, familial hypercholesterolemia, phenotype, LDL cholesterol, polygenic score, single-nucleotide polymorphism

## Abstract

Familial hypercholesterolemia (FH) is the most significant inherited risk factor for coronary heart disease (CHD). Current guidelines focus on monogenic FH, but the polygenic form is more common and less understood. This study aimed to assess the clinical utility of an 8-SNP LDLC polygenic score in a central Romanian cohort. The cohort included 97 healthy controls and 125 patients with premature (P)CHD. The weighted LDLC polygenic risk score (wPRS) was analyzed for associations with relevant phenotypic traits, PCHD risk, and clinical FH diagnosis. The wPRS positively correlated with LDLC and DLCN scores, and LDLC concentrations could be predicted by wPRS. A trend of increasing LDLC and DLCN scores with wPRS deciles was observed. A +1 SD increase in wPRS was associated with a 36% higher likelihood of having LDLC > 190 mg/dL and increases in LDLC (+0.20 SD), DLCN score (+0.16 SD), and BMI (+0.15 SD), as well as a decrease in HDLC (−0.14 SD). Although wPRS did not predict PCHD across the entire spectrum of values, individuals above the 90th percentile were three times more likely to have PCHD compared to those within the 10th or 20th percentiles. Additionally, wPRS > 45th percentile identified “definite” clinical FH (DLCN score > 8) with 100% sensitivity and 45% specificity. The LDLC polygenic score correlates with key phenotypic traits, and individuals with high scores are more likely to have PCHD. Implementing this genetic tool may enhance risk prediction and patient stratification. These findings, the first of their kind in Romania, are consistent with the existing literature.

## 1. Introduction

Coronary heart disease (CHD) is the leading cause of death in industrialized countries and is driven by both genetic and environmental factors. Traditional risk factors only partially explain the variability in disease risk, and genetic studies have confirmed that CHD susceptibility is significantly heritable [1]. Genetic analysis can detect causative mutations, identify pathological pathways, and candidate therapeutic targets. The association of genetic markers with traditional cardiovascular risk factors is clinically significant because they can be measured accurately, are stable over time, need to be determined only once, and allow for the simultaneous evaluation of many genetic characteristics.

Following the Human Genome Project, genome-wide association studies and meta-analyses have identified numerous single-nucleotide polymorphisms (SNPs) and gene mutations linked to elevated blood lipid levels and increased CHD risk, confirming the heritable nature of CHD susceptibility [2,3,4]. Understanding a population’s genetic risk burden can lead to the development of population-specific genetic risk scores, improving CHD prediction and enabling personalized preventive measures. Given the genetic and epidemiological differences in CHD prevalence across European countries [5,6], these scores should be optimized for the targeted populations and validated in prospective cohort studies [7,8,9]. While some genetic risk scores have been validated in large-scale studies [10,11,12,13], the cardiovascular genetic risk profile of the Romanian population is largely unknown. In our earlier research, we sought to investigate monogenic familial hypercholesterolemia (FH) within the Romanian population [14,15]. However, the Randox PCR multiplex FH arrays proved ineffective for this demographic. As a result, we shifted our focus toward exploring polygenic FH. This study aims to assess the genetic risk profile in central Romania by developing a polygenic risk score (PRS) and evaluating its association with relevant cardiovascular risk traits. This may enable better CHD susceptibility assessment, potentially enhancing public health prevention strategies in Romania. This study did not aim to diagnose polygenic HF or to distinguish it from the monogenic form.

## 2. Results

### 2.1. Genetic Screening for FH Mutations

In the overall sample (n = 226), screening with the Randox FH arrays I and II detected four patients with the following mutations: LDLR FH12 c.2054C > T (p.Pro685Leu); APOB FH1 c.10580G > A (p.Arg3527Gln); LDLR FH28 c.1706-10G > A [p.(=)]; and LDLR FH39 c.1618G > A (pAla540Thr) [14]. LDLC was significantly higher in the mutation positive (FH/M+) group compared with PCHD patients that tested negative (*p* = 0.015). After excluding FH/M+ patients, a total number of 97 individuals as the healthy control (HC) and 125 PCHD cases were included in the present study. During statistical analysis, where applicable, PCHD patients were split into two groups: with “definite” clinical FH (dFH, DLCN score > 8, n = 8) and without “definite” clinical FH (nFH, DLCN ≤ 8, n = 117).

It is important to note that, given the poor performance of the Randox FH arrays in our population, we refrained from labeling these participants as FH/M−.

### 2.2. Demographic, Clinical, and Biological Characteristics

Demographic, clinical, and biological characteristics are presented and compared between the three groups in Table 1.

### 2.3. Genetic Analysis and Risk Scores

Seven LDLC-affecting SNPs were tested in both PCHD patients and controls, and both groups were found to be in Hardy–Weinberg equilibrium. Linkage disequilibrium analysis led to the exclusion of APOB rs676210 due to its strong linkage disequilibrium with APOB rs1367117 (*p* < 0.001, D’ = 0.99). The remaining six SNPs, along with two APOE SNPs, were used to calculate the 8-SNP LDLC-affecting PRSs (aPRS and wPRS). Table 2 provides allele frequencies, PRS calculations, and median values across the groups. Comparative allele frequency data from the literature [4,10] are available in Supplementary Table S1.

### 2.4. Establishing the Superior PRS

**Figure 1:**
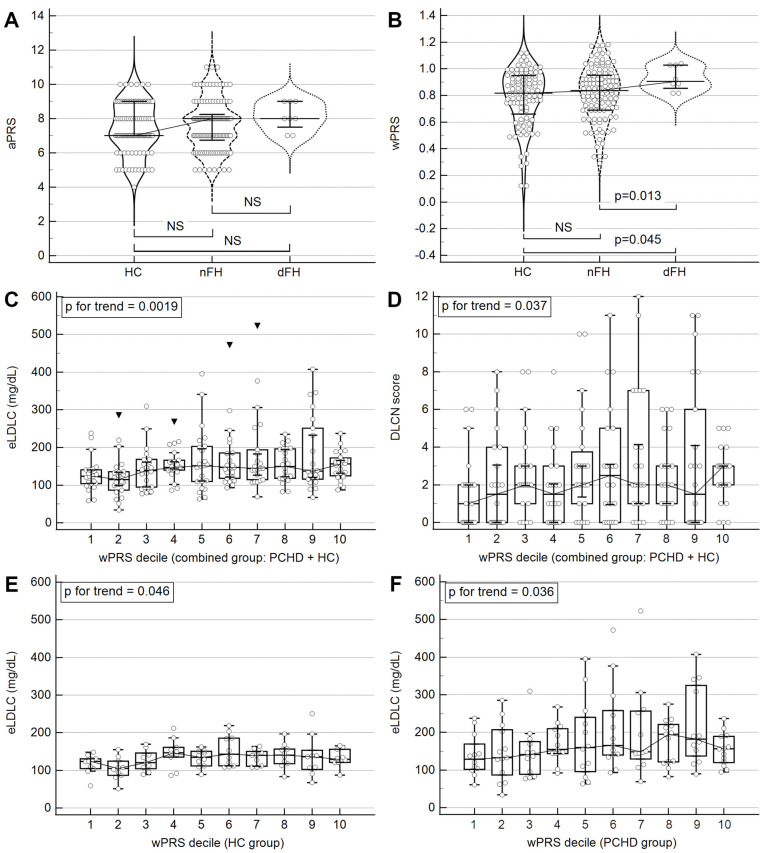
Relationship between PRSs and phenotypic traits: eLDLC and DLCN scores. All figures display median values with error bars representing the interquartile range. (**A**): comparison of median aPRS between HC, nFH, and dFH groups. (**B**): comparison of median wPRS between HC, nFH, and dFH groups. (**C**): trend analysis of eLDLC concentrations based on wPRS decile in the whole cohort. (**D**): trend analysis of DLCN scores based on wPRS decile in the whole cohort. (**E**): trend analysis of eLDLC concentrations based on wPRS decile in the HC group. (**F**): trend analysis of eLDLC concentrations based on wPRS decile in the PCHD group. The trend in (**C**–**F**) was evaluated using the Jonckheere–Terpstra statistical test. Abbreviations: dFH, definite FH (DLCN score > 8); eLDLC, estimated pre-treatment LDL cholesterol; FH, familial hypercholesterolemia; HC, healthy control; nFH, non-definite FH (DLCN score ≤ 8); NS, nonsignificant; PCHD, premature coronary heart disease (patients); PRS, polygenic risk score (additive/a or weighted/w).

Mann–Whitney comparison of PRSs between HC and PCHD showed no significant difference. Subgroup analysis revealed a slightly higher aPRS in nFH and dFH compared to HC, but these differences were not statistically significant (Figure 1A). In contrast, wPRS was significantly higher in dFH (median 0.906) compared to the other groups (nFH: median 0.837; HC: median 0.818; Figure 1B).

Statistical analysis revealed weak but marginally significant correlations between the PRSs and two major phenotypic aspects: eLDLC and DLCN scores. Both aPRS and wPRS correlated better with eLDLC levels than with the DLCN score. However, wPRS showed a stronger correlation and higher statistical significance compared to aPRS (Supplementary Table S2).

We examined risk ratios among PRS deciles for two outcomes: eLDLC > 190 mg/dL and DLCN score > 8. This analysis was conducted separately for the HC and PCHD groups and for the combined group. Due to small decile sizes, the comparisons did not achieve statistical significance (Supplementary Table S3). Additionally, we tested for trends between PRS deciles and eLDLC/DLCN. In the combined group, there were significant and marginally significant increases in eLDLC levels (*p* = 0.031) and DLCN scores (*p* = 0.099) with rising aPRS deciles, respectively. Similarly, a more significant trend was noted with increasing wPRS deciles (Figure 1C,D). In the HC, PCHD, and nFH groups, there were significant or marginally significant upward trends in eLDLC levels, but not in DLCN scores, as the wPRS deciles increased (Figure 1E,F). For the complete analysis, refer to Supplementary Table S4.

The evidence thus far indicated that the wPRS was superior concerning its association with phenotypic aspects. Therefore, subsequent analyses were concentrated on exploring the relationship between wPRS and relevant phenotypic traits.

### 2.5. Phenotypic Traits Associated with wPRS

Principal component analysis demonstrated that wPRS grouped together with eLDLC in the second component, which accounted for 12% of the variability in the data from PCHD patients. For the complete results of the principal component analysis, refer to Supplementary Table S5.

**Figure 2:**
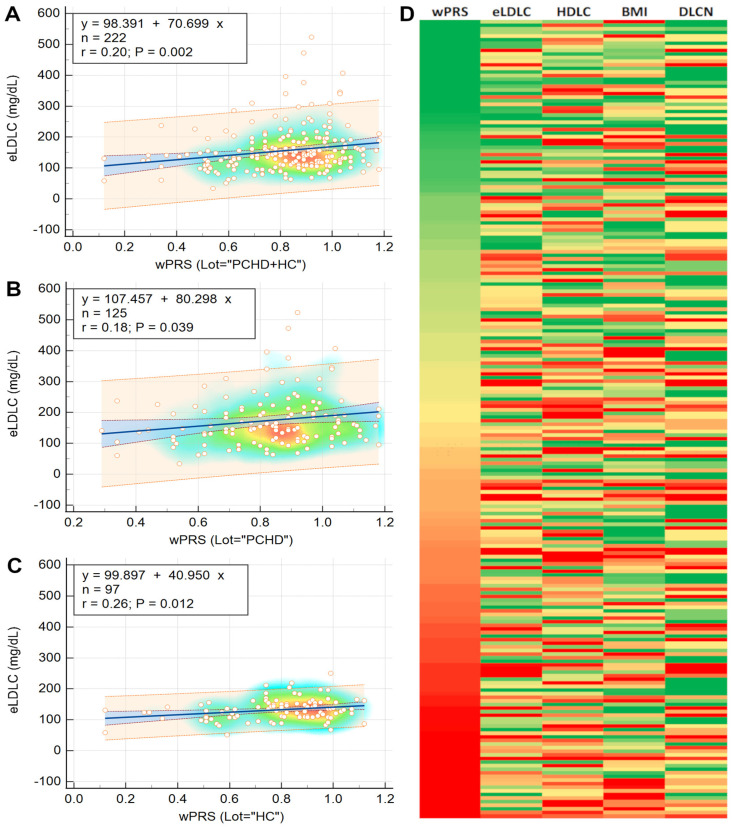
(**A**–**C**) represent eLDLC-wPRS regression equations for the whole cohort, PCHD group, and HC group, respectively. (**D**) illustrates phenotypic trait variations with wPRS: variables are color-coded on a gradient from green (low) to red (high), with the exception of HDLC, which is color-coded from red (low) to green (high). Abbreviations: BMI, body mass index; DLCN, Dutch Lipid Clinic Network (score); eLDLC, estimated pre-treatment LDL cholesterol; HC, healthy controls; PCHD, premature coronary heart disease (patients); wPRS, weighted polygenic risk score.

Regression analysis showed that wPRS can not only be used to predict the concentration of eLDLC in the cohort as a whole, but also in the PCHD and HC subgroups (Figure 2A–C). The associations between the wPRS Z score and the demographic and phenotypic characteristics are presented in Table 3. For a visualization of how eLDLC, HDLC, DLCN score, and BMI (the traits most associated with wPRS) vary with the wPRS, see the heatmap in Figure 2D.

### 2.6. wPRS Association with PCHD

Binomial logistic regression analysis was performed in order to detect potential PCHD predictors. Univariate analysis results are shown in Table 4. Of the two PRSs, only the wPRS showed a marginally significant association with PCHD (*p* < 0.15).

For the multivariate logistic regression analysis, we included variables with significant (*p* < 0.05) or marginally significant (*p* < 0.15) associations with PCHD from the univariate analysis. The DLCN score was excluded due to its tendency to produce non-uniform regression models when combined with other variables, as indicated by the Hosmer–Lemeshow test (*p* < 0.0001). We formulated two predictive models for PCHD: a reduced model (clinical and demographic variables) and a complete model (reduced model plus laboratory variables). The results are presented in Table 5.

**Figure 3:**
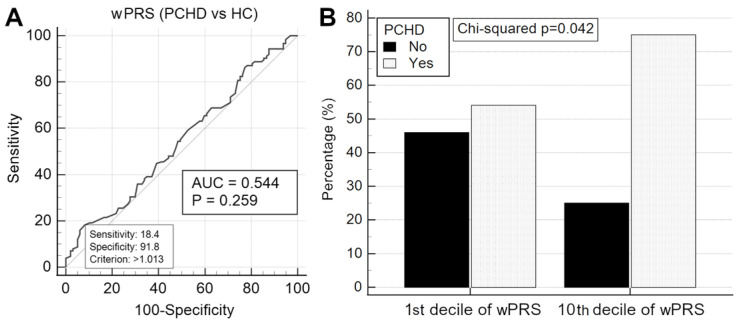
The performance of wPRS in predicting premature coronary heart disease (PCHD). (**A**): ROC analysis to differentiate between HC and PCHD based on wPRS. (**B**): comparison of PCHD prevalence between those with the lowest (first decile) and highest (tenth decile) wPRS scores. Abbreviations: AUC, area under the curve; HC, healthy control; wPRS, weighted polygenic risk score.

We then compared the two models to determine if incorporating the wPRS would enhance their predictive performance. The complete model demonstrated superior predictive power compared to the reduced model (extra sum-of-squares F test, *p* < 0.0001). Adding the wPRS (OR 1.29 (0.92–1.80), *p* = 0.135) only marginally improved the reduced model (AUC values 0.861 (0.808–0.904) vs. 0.855 (0.802–0.899); *p* = 0.131). The lack of discriminatory power of the wPRS was confirmed by ROC analysis (Figure 3A). Logistic regression confirmed that wPRS > 1.013 was not significantly associated with PCHD (OR 1.30 (0.76–2.23), *p* = 0.32). Similarly, the wPRS did not enhance the predictive power of the complete multivariate model.

**Figure 4:**
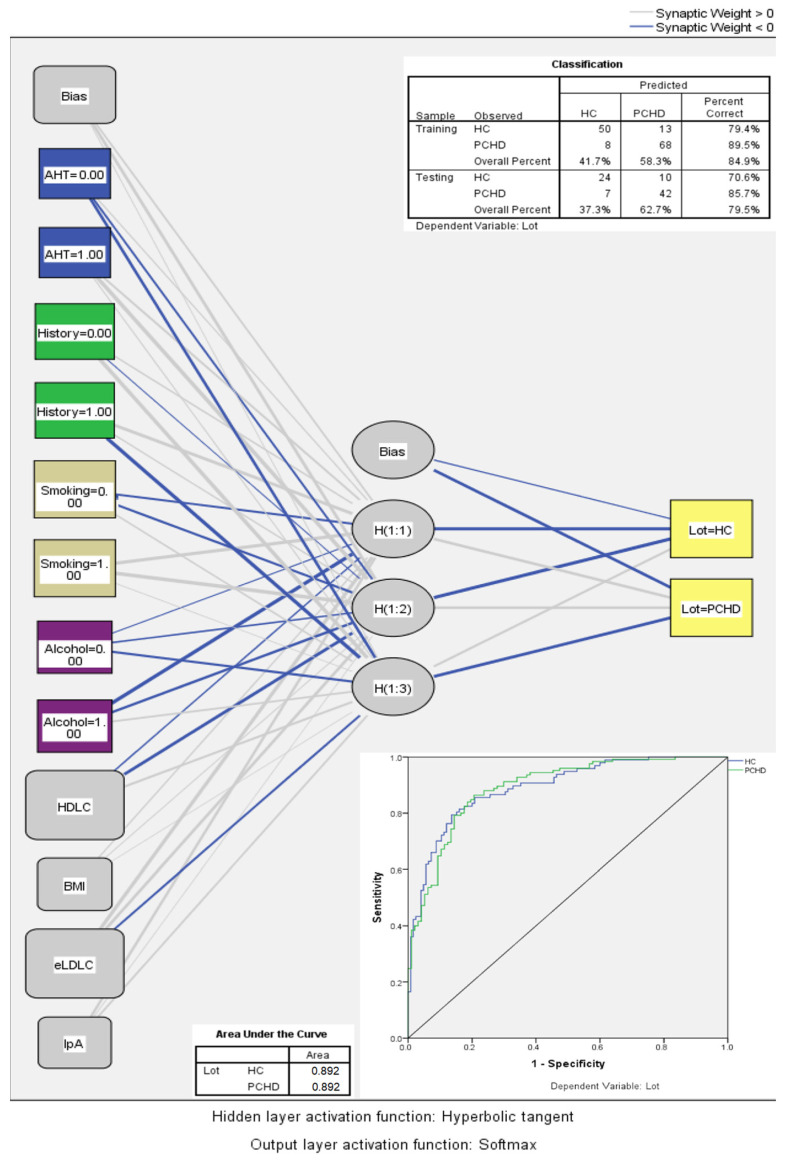
Sample of a multilayer perceptron artificial neural network algorithm for predicting PCHD. “History” stands for family history of CHD. “Alcohol” stands for alcohol consumption. Abbreviations: AHT, arterial hypertension; BMI, body mass index; eLDLC, estimated pre-treatment LDL cholesterol; HC, healthy controls; PCHD, premature coronary heart disease.

**Figure 5:**
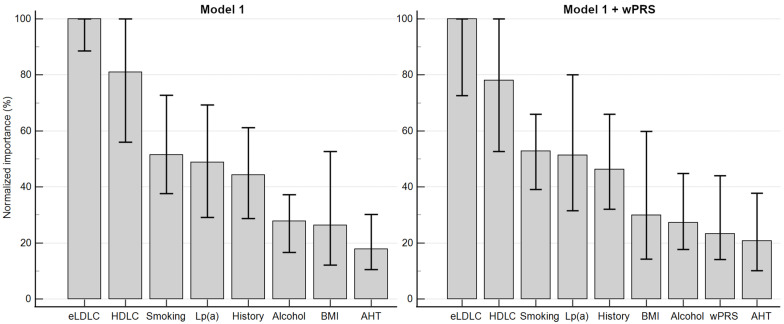
Normalized importance of variables for predicting PCHD. Both models display the average normalized importance calculated after 100 iterations of multilayer perceptron neural network analysis. Medians are shown with error bars representing the 10–90% value interval. “History” stands for family history of CHD. “Alcohol” stands for alcohol consumption. Abbreviations: AHT, arterial hypertension; BMI, body mass index; eLDLC, estimated pre-treatment LDL cholesterol; PCHD, premature coronary heart disease; wPRS, weighted polygenic risk score.

The initial multilayer perceptron model integrated the seven variables from the complete multivariate regression model along with BMI. The subsequent model included these variables alongside wPRS. Each model underwent 100 iterations (Table 6). An illustrative neural network is depicted in Figure 4. Incorporating wPRS did not augment the accuracy of the artificial neural network. The average normalized importances of the variables are hierarchically displayed in Figure 5.

Given that regression and artificial neural network models revealed no independent association between PCHD and wPRS across its entire spectrum, we investigated whether individuals with the highest wPRS were at increased odds of having PCHD compared to those with the lowest wPRS. PCHD was significantly more frequent among those above the 90th percentile of wPRS compared to those within the 10th (Figure 3B) and 20th percentiles. For the complete analysis, refer to Supplementary Table S6.

### 2.7. Association of wPRS with Clinical FH

**Figure 6:**
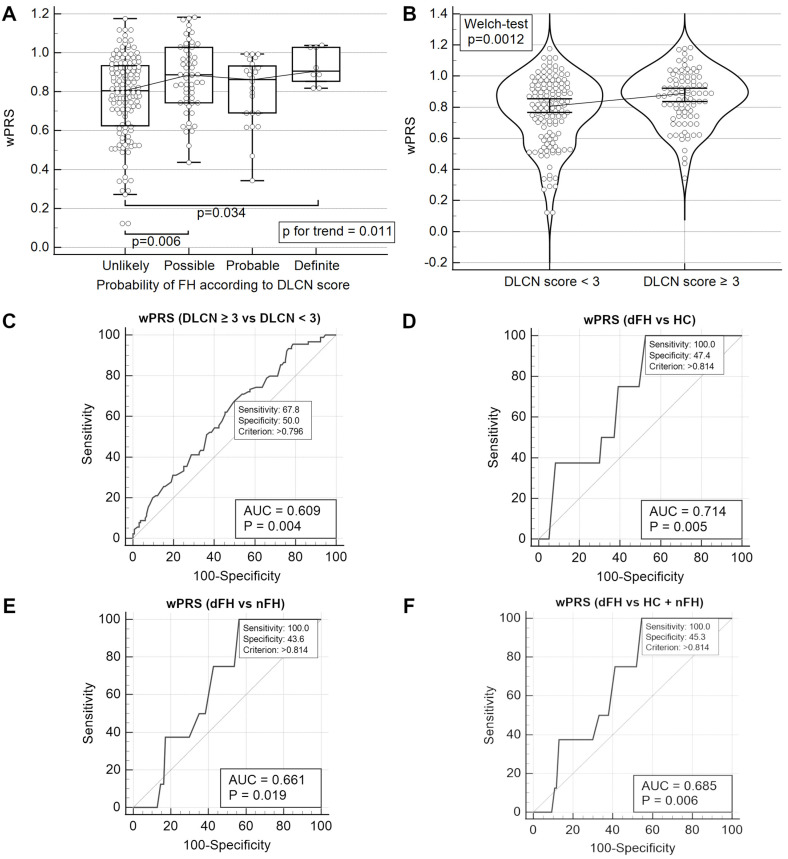
The performance of wPRS in predicting clinical FH. (**A**): whole cohort trend analysis of wPRS based on DLCN score categories. (**B**): whole cohort comparison of wPRS scores between those with “unlikely FH” (DLCN < 3) and the other participants. (**C**): whole cohort ROC analysis to differentiate between those with “unlikely FH” (DLCN < 3) and the other participants, based on wPRS. (**D**): ROC analysis to differentiate between dFH patients and HC based on wPRS. (**E**): ROC analysis to differentiate between dFH and nFH patients based on wPRS. (**F**): ROC analysis to differentiate between dFH patients and the other participants based on wPRS. Classification for DLCN score categories in panel A is as follows: unlikely (DLCN < 3), possible (DLCN 3-5), probable (DLCN 6-8), and definite (DLCN > 8). Abbreviations: AUC, area under the curve; dFH, definite FH (DLCN score > 8); DLCN, Dutch Lipid Clinic Network (score); FH, familial hypercholesterolemia; HC, healthy controls; nFH, non-definite FH (DLCN score ≤ 8); PCHD, premature coronary heart disease (patients); wPRS, weighted polygenic risk score.

Study participants were grouped based on the DLCN score categories for FH probability: unlikely, possible, probable, and definite. Analysis using the Kruskal–Wallis test showed that compared to the “unlikely” category, wPRS was significantly higher in the “possible” and “definite” categories, but not in the “probable” category. Additionally, the Jonckheere–Terpstra test indicated a significant trend, demonstrating that wPRS values increase significantly with each DLCN score category (Figure 6A).

In the literature, it is common practice to classify participants in a dichotomous manner using the DLCN score. Thus, we first divided all study participants into two categories: the two uppermost categories (that is, DLCN score ≥ 6) and the two bottommost categories (that is, DLCN score < 6). We found no difference in wPRS between these two categories.

We further divided the participants into two groups: DLCN score ≥ 3 and DLCN score < 3. Significant differences in wPRS were observed (Figure 6B). ROC analysis further confirmed these results (Figure 6C), while logistic regression indicated that a 1 SD increase in wPRS increased the odds of having a DLCN score ≥ 3 (OR 1.58 (1.17–2.13), *p* = 0.0016).

The last dichotomous model compared DLCN > 8 with DLCN ≤ 8. We tested whether wPRS could discriminate between these categories in the following scenarios: dFH vs. HC (Figure 6D), dFH vs. nFH PCHD patients (Figure 6E), and dFH vs. non-dFH (Figure 6F).

## 3. Discussion

### 3.1. Monogenic FH

FH (OMIM 143890) is a significant public health concern and the most important inherited risk factor for CHD. Despite advances in diagnosis and treatment, CHD remains underdiagnosed and undertreated globally, with varied management practices across countries, including Romania, where CHD prevalence remains high. A 2018 EAS-FHSC survey across 63 countries highlighted the urgent need to increase FH awareness, detection, and management worldwide [18]. Understanding the genetic risk profile of populations is crucial for improving CHD prediction. Efforts such as genetic research and national registries aim to enhance FH awareness and management. In this context, our study represents one of the first initiatives in Romania.

FH is often caused by monogenic mutations in LDLR, APOB, and PCSK9. However, population genetic screening remains challenging due to the thousands of variants identified within these genes. In some regions, monogenic FH rates are high due to dominant founder effects, but globally, FH/M+ rates vary from 15% to 93% [19,20,21,22,23,24,25,26,27,28,29,30,31]. For example, the Randox Biochip Array Technology detected 40 FH-causing mutations in an Oxford cohort with a 71.3% detection rate among known mutation carriers and 7.4% among patients meeting the Simon Broome minimum criteria [32]. In our Romanian cohort (n = 226), using the same technology, we found four mutations, resulting in a 1.77% overall detection rate, 3.10% in PCHD patients, 3.41% in those with a DLCN score > 2, 8.57% in those with a DLCN score > 5, and 20.00% in those with a DLCN score > 8. Using the same Randox arrays, we identified four mutations described in the Results section. Furthermore, we identified and described three pathogenic or likely pathogenic variants in the LDLR gene: LDLRc.1171G > A, LDLRc.1060 + 59A > C, and LDLRc.1413A > T [14]. To our knowledge, this was the first report of these variants in central Romania. Notably, three of these mutations (LDLRc.1618G > A, LDLRc.1060 + 59A > C, and LDLRc.1413A > T) were also found by Vlad et al. in northeastern Romania [33]. Together, these findings suggest that these mutations could form a tailored monogenic panel for screening Romanian patients with clinical FH.

It is important to note that, given the poor performance of the Randox FH arrays in our population (which was a carefully selected cohort with angiographically confirmed PCHD), we recognize that some of the individuals who tested negative may still carry a mutation. Therefore, we refrained from labeling these participants as FH/M− and acknowledge that, under the current circumstances, we were unable to clearly differentiate between the polygenic and monogenic forms of FH.

### 3.2. Genotype–Phenotype Correlation

Genetic testing is the gold standard for diagnosing FH, but its unfavorable benefit/cost ratio limits widespread screening. Thus, clinical scoring systems like DLCN or Simon Broome criteria are commonly used to identify patients likely to have genetic mutations. However, not all clinically diagnosed individuals have detectable mutations and vice versa. In our cohort, the genotype–phenotype relationship varied: of the four FH/M+ patients, one had “unlikely FH”, one had “probable FH”, and two had “definite FH”. Notably, all three patients with novel pathogenic or likely pathogenic variants had “probable FH”. Of the ten patients with “definite FH”, only two were FH/M+. Therefore, in our central Romanian cohort, only 20% of “definite FH” patients were FH/M+, and only 50% of FH/M+ patients had a “definite FH” diagnosis. This discrepancy may be due to the limited mutations tested and the potential inadequacy of the Randox FH arrays for the Romanian population. Nevertheless, the poor genotype–phenotype overlap suggests the possibility of a polygenic cause.

### 3.3. Polygenic FH

Globally, about 60% of patients with elevated LDL-C levels have no known mutation in the major FH-associated genes [10,12]. Although some rare mutations may not be identified, it is believed that most FH/M− cases might be explained by polygenic inheritance, where multiple small-effect LDLC-raising alleles contribute to the phenotype and cardiovascular risk. In our study, 80% of patients with “definite” FH (DLCN > 8) and 96% with “possible” or “probable” FH (DLCN 3-8) were mutation-negative. This indicates that the Randox FH arrays, tailored for UK and Ireland populations, may not be suitable for the Romanian population. Nevertheless, considering 73% of our PCHD cohort developed CHD before age 40 and 62% had a “possible” or “probable” FH diagnosis, we explored polygenic hypercholesterolemia in our cohort.

The concept of a genetic score consistent with polygenic FH involves integrating multiple genetic variants associated with elevated cholesterol levels to predict an individual’s genetic predisposition to high cholesterol and CHD. Over the past decade, numerous studies have tested various PRSs, consistently demonstrating their association with LDLC concentrations [10,11,12,34,35,36,37,38,39,40,41]. Significant progress has been made in identifying SNPs and gene mutations associated with elevated cholesterol levels and increased CHD risk. A 2010 meta-analysis of 46 lipid genome-wide association studies, involving over 100,000 individuals of European descent, identified 95 loci associated with serum lipid traits, including LDLC, HDLC, and TG concentrations [4]. This laid the foundation for LDLC PRSs, focusing on alleles with significant effects on LDLC levels. In 2013, Talmud et al. published an influential study proposing a 12-SNP PRS to differentiate between monogenic and polygenic FH [10]. In 2015, de Vries et al. tested a 152-SNP PRS, which predicted the incidence of CHD, but failed to improve CHD risk prediction [34]. Further, Futema et al. refined a 6-SNP PRS which performed comparably to the original 12-SNP score [12]. More recently, the same research group reported that the 12-SNP PRS varies across ethnicities, underscoring the importance of developing population-specific PRSs [11]. Despite these efforts, knowledge about the cardiovascular polygenic risk profile remains limited in many countries, including Romania.

In this study, we utilized an LDLC PRS based on eight SNPs validated in European populations [10,11,12,34,35,38,41]. We developed both additive and weighted variants of the score, with the wPRS showing superior performance across all metrics. The allele frequencies observed in our central Romanian population were comparable to those reported in a large European cohort [4] and UK population [10] (Supplementary Table S1).

### 3.4. LDLC Polygenic Score Is Associated with Phenotypic Traits

With variable penetrance, FH displays significant phenotypic variability within and between families, influenced by factors like genetic modifiers, lifestyle, and environment. This heterogeneity leads to diverse genotype–phenotype correlations among individuals with FH. Here, we assessed the association of an 8-SNP LDLC PRS with phenotypic traits relevant to atherosclerotic cardiovascular disease. Given its superior performance, we focus exclusively on wPRS in our discussion below.

The wPRS showed statistically significant correlations with eLDLC levels across PCHD patients, the HC group, and the combined cohort. Similarly, a significant association was observed between wPRS and the DLCN score in the overall cohort, with marginal significance noted in the PCHD and HC groups (Supplementary Table S2). These positive correlations were reinforced by a notable trend of increasing eLDLC levels and DLCN scores across wPRS deciles (Figure 1C–F). Simple linear regression analyses demonstrated that eLDLC levels could be predicted based on wPRS values across the entire cohort and within PCHD and HC subgroups individually (Figure 2A–C). Moreover, principal component analysis identified that the second principal component, consisting of eLDLC and wPRS, explained 12% of the variability in PCHD patient data (Supplementary Table S5).

Quantifying the association of wPRS with phenotypic traits, regression analysis revealed that a +1 SD increase in wPRS was linked to a 36% higher likelihood of having eLDLC levels above 190 mg/dL. This increase in wPRS was also associated with significant rises in eLDLC levels (+0.20 SD or +0.36 mmol/L or +14 mg/dL), DLCN score (+0.16 SD or 0.43 points), and BMI (+0.15 SD or +0.70 kg/m^2^), alongside a notable decrease in HDLC (−0.14 SD or −0.05 mmol/L or −2 mg/dL). Comparable findings were reported by Talmud et al., showing that a +1 SD increase in the PRS correlated with a +0.33 mmol/L (+12.7 mg/dL) rise in LDLC levels [10]. Wu et al. similarly found significant associations, where a +1 SD increase in their PRSs was associated with LDLC level increases of +0.34 mmol/L (+13.1 mg/dL) and +0.42 mmol/L (+16.2 mg/dL) in mutation carriers and noncarriers, respectively [40]. These results align with other studies reporting positive associations between PRSs and LDLC concentrations [11,35,36,38]. Thus, our findings, consistent with similar studies, suggest a robust association between wPRS and clinically relevant phenotypic traits, mainly lipoprotein concentrations, in the central Romanian population. This association fits within the broader context of cardiovascular risk factors for CHD in which LDLC plays a central role.

### 3.5. Elevated LDLC Polygenic Score Is Associated with Premature Coronary Heart Disease

Given that our patients had an unequivocal diagnosis of CHD based on angiographic coronary evidence, we tested whether wPRS could discriminate PCHD. Multiple statistical analyses yielded negative results: logistic regression (Table 4), ROC curve analysis (Figure 3A), no improvement to multivariate models (Table 5), and no improvement to multilayer perceptron artificial neural network models (Table 6). However, with regard to PCHD discrimination, it is worth noting that the complete regression model and the multilayer perceptron neural network model performed very well at identifying PCHD without the polygenic score, yielding very similar performances (AUC values of 0.895 and 0.898, respectively).

When it comes to the role of PRSs in predicting CHD, data from the literature are contradictory. Some studies have reported a significant association between PRSs and CHD incidence [34,40,42,43], while others have found no relationship [38,44,45]. These disparities may be explained by the various PRS models employed as well as differences in cohort sizes. Given that in the present study, no association between wPRS and PCHD was found across the entire spectrum of wPRS values, we investigated whether individuals with the highest wPRS values were at increased odds of having PCHD compared to those with the lowest wPRS values.

Our analysis revealed that individuals with wPRS above the 90th percentile are about three times more likely to have PCHD compared to those within the 10th percentile (Figure 3B) and 20th percentile. This association was also noted across all other percentiles, with *p*-values consistently close to the significance threshold (Supplementary Table S6), indicating that a wPRS above the 90th percentile is a potential risk factor for PCHD. These findings align with evidence from the literature showing that polygenic hypercholesterolemia, unlike monogenic hypercholesterolemia, is not a dichotomous diagnosis but rather exists on a continuous scale that confers CVD risk in a dose-dependent manner [46]. Thus, as discussed later, these findings may hold clinical significance for refining risk stratification among individuals at risk, but who have yet to develop symptomatic CHD.

### 3.6. LDLC Polygenic Score Is Associated with Clinical FH Phenotype

In this study, we reported that wPRS was significantly higher in the PCHD subgroup with a “definite” clinical diagnosis of FH compared to other patients and particularly compared to healthy individuals (Figure 1B). Additionally, we observed a weak but statistically significant positive correlation between wPRS and DLCN score (Supplementary Table S2). Analysis of the entire cohort revealed a significant trend: an increase in wPRS decile was associated with an increase in DLCN score (Figure 1D), with regression analysis indicating that a +1 SD increase in wPRS corresponded to a +0.16 SD increase in DLCN score. These findings suggest that the DLCN score increases with wPRS.

In examining the predictive value of wPRS for FH likelihood, we observed an increasing trend of wPRS across DLCN categories (Figure 6A). Additionally, wPRS effectively differentiated between DLCN categories. For instance, wPRS was higher in the DLCN ≥ 3 category compared with DLCN < 3. ROC curve analysis indicated that wPRS > 0.796 could distinguish between these categories with 67.8% sensitivity and 50.0% specificity (Figure 6B,C). Moreover, wPRS > 0.814, that is, >45th percentile, could distinguish “definite” from “non-definite” clinical FH with 100% sensitivity and just under 50% specificity across the cohort or its subgroups (Figure 6D–F). The high sensitivity of this test implies virtually no false negatives, ensuring the identification of all individuals with “definite” clinical FH. Since the PRS is a genetic measure that remains constant throughout an individual’s lifetime, it may have the potential to enable an early identification of at-risk individuals even before the FH phenotype manifests. While the test’s modest specificity of 50% may lead to a higher rate of false positives, this is not necessarily problematic because the result should always be interpreted in the context of the patient’s history, clinical presentation, and laboratory findings, which are the definitive criteria for diagnosing FH. Therefore, for patients of a mature age, where the FH phenotype would be expected to have already manifested, any discrepancies between the PRS and clinical or laboratory findings should raise the suspicion of a false positive. Conversely, if the test is performed earlier in life for at-risk patients, a false positive would once again not have negative consequences. Instead, it would lead to enhanced surveillance, enabling timely intervention and management should the patient progress towards an FH phenotype—a scenario statistically occurring in one out of two surveilled patients. Overall, in this context, the test would not be intended for diagnosis, but could be highly applicable for screening purposes, facilitating the early detection of a silent and at-work genetic risk that may eventually lead to the development of an FH phenotype.

### 3.7. Clinical Relevance and Implementation of LDLC Polygenic Score

Detecting FH-causing mutations is essential for definitive FH diagnosis and the effective screening of first-degree relatives, achieving a 50% detection rate [47]. Additionally, genetic testing is crucial for estimating FH prevalence and identifying common causative mutations in specific populations. Therefore, established national programs and protocols for monogenic FH facilitate the identification of mutations and cascade testing of patients’ families once a mutation is found [47]. However, a monogenic cause does not explain all FH cases, with reported rates of FH/M+ in canonical genes ranging from 15% to 93% [19,20,21,22,23,24,25,26,27,28,29,30,31]. For most FH/M− cases, a polygenic cause is suggested by significantly higher LDLC PRSs in FH/M− compared to healthy individuals [10,35,36,41,48,49] and FH/M+ patients [10,36,41,48]. Despite this, current clinical guidelines do not provide recommendations on whether and how PRSs should be used [46]. The literature reveals significant heterogeneity in the study of PRSs. The alleles included vary widely, from six to millions, making comparisons difficult. Proposed percentile cut-offs for high polygenic scores range from the 25th to the 95th percentile, though most studies use the 75th to 90th percentiles. Additionally, PRS determination is limited to a few clinics and laboratories, hindering widespread adoption. These inconsistencies and limitations highlight the need for collaboration, standardization, and validation through prospective studies.

One widely used PRS is the 12-SNP model proposed by Talmud et al. [10], which was later reduced to a 6-SNP version with similar performance [12]. Both the 12-SNP and 6-SNP PRSs have gained popularity and have been validated in various cohorts in Europe [25,35,48] and globally [36,37,49]. Other studies have reported similar results using “intermediate” PRSs consisting of a number of SNPs between 6 and 12 [41,50]. In the present study, we have also employed an “intermediate” PRS consisting 8 out of the 12 SNPs initially described by Talmud et al. [10]. Expanding the number of analyzed SNPs can enhance the performance of these scores [51], with more complex models explaining up to 21% [40] and even 29% [52] of the variance in LDLC levels. However, for successful clinical implementation, a realistic balance between a score’s performance and its feasibility must be achieved, considering the benefit/cost ratio and the existing infrastructure and personnel training. The simpler yet effective 12- or 6-SNP scores are advantageous as they can be assessed using PCR-based methods, which have become more prevalent and accessible worldwide due to the COVID-19 pandemic. In contrast, PRSs requiring large numbers of SNPs are limited by the current cost and inaccessibility of genome sequencing for many clinical laboratories.

Considering all this, a crucial question remains: why should polygenic scores warrant generalized adoption? What are the benefits such scores would bring, and at what cost? In this context, we aim to briefly highlight some of the most significant advantages of incorporating the assessment of an LDLC polygenic score into the mainstream practice of cardiovascular risk assessment.

First, LDLC polygenic risk score (PRS) testing is a “once-in-a-lifetime” investigation that is both cost-effective and widely accessible. Implementing a PRS could help validate polygenic FH as a genetically proven diagnosis. In patients with an FH phenotype but a low PRS, it may prompt reconsideration of a monogenic cause, potentially involving a rare or unidentified mutation. Even for individuals with known monogenic FH, PRSs could assist in risk stratification, as those with both monogenic and polygenic FH face the highest cardiovascular risk [35,44]. Research has shown that a high PRS is linked to an elevated cardiovascular risk in individuals with borderline LDL-C, compared to those with similar LDL-C levels but without an elevated PRS [53]. Thus, PRSs could aid in risk assessment even in individuals without an FH phenotype. The broader implementation of PRSs could refine clinical trials and redefine personalized lipid-lowering therapies, as evidence suggests that PRSs, even beyond LDL-C, contribute to further CHD risk [53,54,55]. Furthermore, PRS influences the efficacy of lipid-lowering treatments, with individuals having higher scores showing greater benefit from therapies such as statins [55], alirocumab [54], and evolocumab [56]. Lastly, PRS use has been shown to improve patient adherence to lipid-lowering therapies [57]. In light of these potential advantages, the use of LDL-C PRS in clinical practice deserves further attention and support.

### 3.8. Strengths and Limitations

This study had several limitations. First, the cohort size was relatively small compared to similar studies, potentially reducing the statistical power to detect subtle genotype–phenotype relationships. Second, the Randox FH arrays used may not have been optimal for the Romanian population, possibly missing some monogenic FH cases. Third, over 90% of the cohort was from central Romania, limiting the generalizability of the findings to the whole Romanian population. Fourth, due to biological variation and reliance on self-reported statin therapy data, estimated pre-treatment LDLC levels may be inaccurate, thereby also affecting the DLCN score. Finally, Lp(a) interference in LDLC assays could lead to inaccuracies in LDLC measurements, particularly in individuals with high Lp(a) levels, also affecting the DLCN score.

This study also had several strengths. Firstly, all patients included in the study had angiographically confirmed CHD, a criterion not consistently met in similar studies. Despite the relatively small cohort size, our main findings are consistent with those of comparably larger studies in the literature, which, combined with a thorough and multimodal statistical analysis, compensates to some degree for the smaller cohort. Additionally, this study is one of the pioneering efforts to address the issue of FH in Romania and, to our knowledge, the first to describe the applicability of a polygenic score in the Romanian population, thereby advancing the understanding of this topic within an understudied European population.

## 4. Materials and Methods

### 4.1. Study Design and Selection of Participants

This study is part of a larger study approved by the Ethics Committee of the Emergency Institute for Cardiovascular Diseases (486/19 January 2017) and all procedures were performed according to the Declaration of Helsinki. All participants gave written, informed consent for study participation. This study was designed as an unmatched case–control study and participants were enrolled over a one-year period either as cases or controls. The case group consisted of consecutive patients from the Emergency Institute for Cardiovascular and Transplant Diseases of Târgu Mureș, Romania, who met the following inclusion criteria: (1) over 18 years of age, (2) diagnosed CHD defined as positive coronary angiography with significantly obstructive plaques (>50% stenosis) on at least one major coronary artery, (3) premature CHD defined as age at CHD diagnosis between 18 and 50 years for men and 18 and 55 for women, and (4) chronic statin treatment before study participation. All participants were tested for FH-causing mutations using the Randox PCR multiplex FH arrays I and II, which tested for the presence of 40 frequent FH-causing mutations in Caucasians within the low-density lipoprotein receptor (LDLR), apolipoprotein B (ApoB), and proprotein convertase subtilisin/kexin type 9 (PCSK9) genes [14,15]. Only cases negative for the FH mutation screening were included in the present analysis. At the time of enrollment, most cases and some of the controls were under treatment with atorvastatin, rosuvastatin, or simvastatin either as a stand-alone therapy or in combination with fenofibrate or ezetimibe. The control group consisted of apparently healthy subjects with no personal history of cardiovascular disease. Exclusion criteria for both cases and controls were as follows: (1) below 18 years of age; (2) any disease known to affect lipid metabolism, such as diabetes mellitus, chronic kidney disease, chronic liver disease, hypo- or hyperthyroidism, etc.; and (3) a positive result to the FH mutation screening.

### 4.2. Data Collection from Participants

Upon enrollment, all participants were thoroughly interviewed to obtain demographic data and a full medical history focused on cardiovascular risk factors and diseases. A physical examination was performed, anthropometric data were collected, and blood pressure was measured. If available, previous laboratory results were also considered. Data on current lipid-lowering treatment (statin type and dose) was used to estimate the LDLC level prior to statin treatment—eLDLC [16]. Using the eLDLC, a Dutch Lipid Clinic Network (DLCN, without genetic items) score for FH was calculated. Lifestyle factors were self-reported via a written form. After statistical processing of the raw data, smoking status was defined as any participant who is currently a smoker or ex-smoker. Also, alcohol consumption was defined as those who answered that they drink alcohol regularly.

### 4.3. Extended Lipid Profile Analysis

After an overnight fast, peripheral blood was collected in EDTA and serum separator tubes. Serum concentrations of total cholesterol, high-density lipoprotein cholesterol (HDLC), LDLC, and triglycerides were measured on a Cobas Integra 400 plus analyzer (Hoffmann-La Roche, Basel, Switzerland) according to the manufacturer’s instructions, using commercially available kits (Roche, REFs 03039773, 04399803, 07005717, and 20767107, respectively). Non-HDLC was calculated as the difference between total cholesterol and HDLC.

Plasma from EDTA tubes was separated and apolipoproteins AI, AII, B, E, and lipoprotein(a) were measured by nephelometry on a BN ProSpec analyzer (Siemens Healthineers, Munic, Germany) according to the manufacturer’s instructions, using commercially available kits (Behring Diagnostics, London; REFs OUED15, OQBA09, OSAN15, OQDL09, and OQHL11, respectively).

### 4.4. Genetic Analysis and Risk Scores

Whole blood from EDTA tubes was used for DNA purification with the PureLink Genomic DNA Mini Kit (Thermo Fisher Scientific, Aalst, Belgium; Cat. No. K182002) according to the manufacturer’s instructions. SNP genotyping analysis was performed on a 7500 Fast Dx Real-time PCR system (Applied Biosystems, Rotkreuz, Switzerland) and included APOE gene polymorphisms (C112R, R158C) and seven of the most frequent gene variants identified to affect LDLC concentration [10,11,12,13]. The following commercially available SNP genotyping assays from Thermo Fisher Scientific were used (Cat. No. 4351379): APOE rs429358 (C___3084793_20), APOE rs7412 (C____904973_10), APOB rs676210 (C___3216558_10), APOB rs1367117 (C___3216553_20), PCSK9 rs2479409 (C___2018190_10), ABCG8 rs4299376 (C__26135637_10), LDLR rs6511720 (C__34514854_10), CELSR2 rs629301 (C____972957_1_), and HFE rs1800562 (C___1085595_10).

During statistical analysis (see below), one of the seven studied SNPs (APOB rs676210) was excluded. Based on the APOE genotype (two SNPs) and the remaining six SNPs selected from the literature, two 8-SNP “LDL cholesterol-affecting” polygenic risk scores (PRSs) were designed: an additive PRS (aPRS) and a weighted PRS (wPRS). The aPRS was defined as the sum of all detected risk (LDLC-raising) or protective (LDLC-lowering) alleles. The wPRS was defined as the sum of products between each detected risk/protective allele and its corresponding beta-coefficient (weight), according to the literature [4,10,11,12,13,17]. Details on how the two PRSs were calculated are found in Table 2.

### 4.5. Statistical Analysis

Throughout the study, various statistical comparisons were performed between groups. The Chi-squared test was used for nominal variables. For continuous variables, the independent samples *t*-test (or Welch test) and the Mann–Whitney U test were used, depending on the statistical distribution and variance of the datasets. The statistical distribution was tested using the Kolmogorov–Smirnov test and the variance was tested with the F test. Trends associated with the variation of the wPRS were tested using the Jonckheere–Terpstra test. In the present study, statistical significance was considered when *p* ≤ 0.05. *p* values between 0.05 and 0.15 were considered marginally significant and may also be reported. Unless not applicable, only two-tailed *p* values were reported.

Deviation from Hardy–Weinberg equilibrium for the seven studied SNPs was assessed using the Chi-squared test in both the PCHD and the control groups. Linkage disequilibrium was also evaluated for correlated gene polymorphisms using the D coefficient. In order to identify the superior PRS, correlations between each of the two PRSs (additive and weighted) and phenotypic aspects (eLDLC level and DLCN score) were tested using Spearman’s correlation.

The wPRS was also standardized by its mean and SD (wPRS Z score) and further used to test for associations with demographic, clinical, and laboratory variables. Also, the association of several variables (wPRS Z score included) with PCHD was tested in both univariate and multivariate models. The association of the wPRS with a clinical diagnosis of FH, based on DLCN score, was also tested. For these investigations, we used the following statistical tests: univariate and multivariate binomial logistic regression, simple linear regression, and ROC curves. The goodness-of-fit of the regression models was tested with the Hosmer–Lemeshow test. Pairwise comparison of ROC curves for nested models was performed using the extra sum-of-squares F test.

Factor analysis (PCA—principal component analysis with Varimax rotation and Kaiser normalization) was conducted to reduce the initial 11 variables to a smaller number of factors that explain a significant proportion of the total variance (at least 60%). This analysis aimed to identify how these variables clustered together and to determine which variables most effectively explained the overall variance among PCHD patients.

For the prediction of PCHD, apart from multivariate regression models, we employed an artificial neural network approach using a multilayer perceptron model. The data were partitioned with 60% allocated to the training set and 40% to the testing set. The model settings included a standardized rescaling method for covariates and the number of possible units in the hidden layer ranging from 1 to 50. The network architecture was automatically selected by SPSS software version 20, resulting in the following parameters: six hidden layers (excluding the bias unit) with a Hyperbolic tangent activation function and two output layers with a Softmax activation function and a Cross-entropy error function. Due to the randomization of participants into the training and testing partitions, the neural network’s structure and performance are unlikely to be identical upon consecutive tests. This variability is particularly relevant given the relatively small cohort size in this study, compared to the large datasets (“big data”) typically used for such neural networks. Thus, to obtain a more reliable estimate of the neural network’s accuracy in correctly classifying participants into the HC or PCHD groups, we conducted 100 iterations of the multilayer perceptron model and averaged the results, including the percentage of correct classifications, AUC values, and the normalized importance of variables.

## 5. Conclusions

While the diagnosis and management of monogenic FH have been well established, polygenic hypercholesterolemia has yet to be addressed by current guidelines, despite increasing evidence that small-effect LDLC-increasing variants can collectively result in an FH phenotype similar to that of the monogenic form. In a pioneering study on the Romanian population, we demonstrated that an 8-SNP LDLC polygenic score correlates with phenotypic aspects and that individuals with high scores may be at increased risk of premature coronary heart disease. Moreover, the polygenic score may serve as an effective screening tool for the early identification of individuals at risk of developing an FH phenotype. Our findings align in all major aspects with similar studies conducted in other populations across Europe and globally. In conclusion, we have outlined ten potential advantages of integrating LDLC polygenic scores into mainstream cardiology practice. However, the successful implementation of LDLC polygenic scores requires national and international collaboration, standardization, and validation in prospective studies, taking into account each population’s genetic specificity—an endeavor that remains elusive at the time of writing this paper.

## Figures and Tables

**Table 1:** Demographic, clinical, and laboratory characteristics compared between groups.

	HC (n = 97)	nFH (DLCN ≤ 8, n = 117)	HC × nFH *p*-Value	dFH (DLCN > 8, n = 8)	HC × dFH *p*-Value	nFH × dFH *p*-Value
Age (years, median (IQR))	47 (43–51)	50 (46–53)	0.0012	55 (48–57)	0.0195	-
CHD onset (n, %)	0 (0.0%)	117 (100.0%)		8 (100.0%)		
21–30 years		19 (16.2%)		1 (12.5%)		
31–40 years		68 (58.1%)		3 (37.5%)		
41–50 years		30 (25.7%)		4 (50.0%)		
Male (n, %)	77 (79.4%)	96 (82.0%)	-	5 (62.5%)	-	-
Family history of CHD (n, %)	8 (8.2%)	41 (35.0%)	<0.0001	4 (50.0%)	0.0004	-
Smoker/ex-smoker (n, %)	32 (32.9%)	92 (78.6%)	<0.0001	2 (25.0%)	-	0.0007
Alcohol consumption (n, %)	36 (37.1%)	30 (25.6%)	0.07	0 (0.0%)	0.0344	0.10
Arterial hypertension (n, %)	22 (22.7%)	55 (47.0%)	0.0002	3 (37.5%)	-	-
Body mass index (kg/m^2^, median (IQR))	27.5 (25.4–30.1)	29.4 (26.2–31.8)	0.0097	30.3 (23.6–31.2)	-	-
Lipid-lowering treatment (n, %)	8 (8.2%)	80 (68.3%)	<0.0001	8 (100.0%)	<0.0001	0.059
Total cholesterol (mg/dL, median (IQR))	195.5 (173.5–219.1)	156.7 (134.3–183.8)	<0.0001	257.0 (241.1–278.7)	0.0019	<0.0001
LDL cholesterol (mg/dL, median (IQR))	127.3 (107.8–147.9)	100.7 (77.7–122.8)	<0.0001	191.3 (180.3–222.3)	0.0001	<0.0001
eLDL cholesterol ^1^ (mg/dL, median (IQR))	130.7 (108.4–151.9)	146.9 (113.7–197.4)	0.0017	385.8 (343.0–439.5)	<0.0001	<0.0001
HDL cholesterol (mg/dL, median (IQR))	46.8 (39.0–59.5)	38.9 (33.8–47.0)	<0.0001	37.3 (35.4–52.3)	-	-
Non-HDL cholesterol (mg/dL, median (IQR))	145.4 (125.4–170.2)	113.7 (92.1–146.7)	<0.0001	222.0 (193.1–232.8)	0.0018	<0.0001
Triglycerides (mg/dL, median (IQR))	118.5 (79.2–172.2)	133.2 (104.7–183.1)	0.0252	175.6 (109.1–277.5)	-	-
Apo-AI (g/L, median (IQR))	1.240 (1.120–1.390)	1.140 (0.974–1.310)	0.0009	1.215 (0.976–1.390)	-	-
Apo-AII (g/L, median (IQR))	0.353 (0.318–0.411)	0.316 (0.282–0.353)	<0.0001	0.325 (0.297–0.384)	-	-
Apo-B (g/L, median (IQR))	0.868 (0.749–0.995)	0.811 (0.656–0.945)	0.0238	1.260 (1.155–1.390)	0.0005	0.0002
Apo-E (g/L, median (IQR))	0.039 (0.034–0.047)	0.036 (0.032–0.043)	0.0274	0.049 (0.040–0.058)	0.0461	0.0082
Lipoprotein(a) (mg/dL, median (IQR))	9.7 (9.7–16.0)	9.7 (9.7–29.8)	-	9.8 (9.7–24.9)	-	-
DLCN score ^2^ (n, (IQR))	0 (0–1)	3 (2–5)	<0.0001	11 (10–11)	<0.0001	<0.0001
Unlikely FH (n, %)	92 (94.8%)	40 (34.2%)		0 (0.0%)		
Possible FH (n, %)	5 (5.2%)	53 (45.3%)		0 (0.0%)		
Probable FH (n, %)	0 (0.0%)	24 (20.5%)		0 (0.0%)		
Definite FH (n, %)	0 (0.0%)	0 (0.0%)		8 (100.0%)		

Demographic, clinical, and laboratory data collected from premature coronary heart disease patients (PCHD; n = 125) and healthy control participants (HC; n = 97). PCHD patients were divided into two categories based on DLCN score: dFH (definite clinical FH, DLCN score > 8; n = 8) and nFH (non-definite clinical FH, DLCN score ≤ 8; n = 117). For a statistical comparison of means/medians, the independent samples *t*-test or the Mann–Whitney test were used, respectively. Only *p* values ≤ 0.10 are reported. ^1^ Estimation of pre-treatment LDLC concentration was based on statin type and dose, according to [16]. ^2^ The Dutch Lipid Clinic Network (DLCN) score for FH was calculated based on the estimated pre-treatment LDLC concentration (no genetic criteria included).

**Table 2:** Allele frequency and calculation of the 8-SNP LDLC polygenic risk scores.

Reference SNP Cluster ID	Gene	Minor Allele (m)	Common Allele (M)	Contrib. to aPRS (m/M)	Contrib. to wPRS (m/M)	HC (n = 97)	nFH (n = 117)	dFH (n = 8)
						Minor Allele Frequency		
rs6511720	LDLR	T ^1^	G *	0/+1	0/+0.180	0.118	0.068	0.000
rs629301	CELSR2	G ^1^	T *	0/+1	0/+0.146	0.221	0.196	0.000
rs1367117	APOB	A *	G	+1/0	+0.105/0	0.345	0.303	0.312
rs4299376	ABCG8	G *	T	+1/0	+0.071/0	0.350	0.359	0.250
rs1800562	HFE	A ^1^	G *	0/+1	0/+0.057	0.031	0.004	0.000
rs2479409	PCSK9	G *	A	+1/0	+0.052/0	0.350	0.363	0.437
rs429358	APOE	C	T	-	-	0.118	0.128	0.062
rs7412	APOE	T	C	-	-	0.072	0.051	0.000
APOE genotype						APOE genotype frequency		
ε2ε2		-	-	−2	−0.800	0.000	0.000	0.000
ε2ε3		-	-	−1	−0.400	0.103	0.102	0.000
ε2ε4		-	-	0	−0.270	0.041	0.000	0.000
ε3ε3		-	-	0	0	0.660	0.719	0.875
ε3ε4 *		-	-	+1	+0.130	0.196	0.162	0.125
ε4ε4 *		-	-	+2	+0.260	0.000	0.017	0.000
8-SNP LDLC polygenic risk score (PRS)						Median PRS (IQR)		
Additive (a)PRS						7.0 (7.0–9.0)	8.0 (6.7–8.2)	8.0 (7.5–9.0)
Weighted (w)PRS						0.818 (0.661–0.949)	0.837 (0.691–0.953)	0.906 (0.853–1.028)

Analyzed polymorphisms, allele frequency, and calculation of the PRSs for the three investigated groups: HC—healthy control (n = 97); nFH—PCHD patients with non-definite clinical FH (DLCN ≤ 8; n = 117); dFH—PCHD patients with definite clinical FH (DLCN > 8; n = 8). Contributions to aPRS are based on the effects of each allele on the LDLC level (protective: −1; no effect: 0; risk: +1) and aPRS is dimensionless. Contributions to wPRS are based on weights (effect sizes) derived from a large meta-analysis of genome-wide association studies (Teslovich et al., 2010) and are expressed in mmol/L [4]. APOE genotype is based on the two APOE SNPs and APOE weights, expressed in mmol/L, which are based on the research of Bennet et al. (2007) [17]. * Risk (LDLC-raising) alleles (or APOE genotypes). Abbreviations: IQR—interquartile range; FH—familial hypercholesterolemia; PCHD—premature coronary heart disease. ^1^ Based on Teslovich et al. (2010) [4], these minor alleles are considered protective (LDLC-lowering), but their effects are nullified in this study for practical reasons. Instead, weights are assigned to the corresponding common alleles, which are then regarded as risk alleles.

**Table 3:** Associations between wPRS Z score and the demographic and phenotypic characteristics.

Variable	Measurement Level	Standardized β Coefficient or Odds Ratio with (95% CI) and *p*-Value			
		Combined Group (n = 222)	HC (n = 97)	PCHD (n = 125)	PCHD nFH (n = 117)
Age ^1^	≤45 years	Ref	Ref	Ref	Ref
	>45 years	1.01 (0.76–1.34), *p* = 0.933	0.82 (0.54–1.25), *p* = 0.360	1.14 (0.75–1.73), *p* = 0.526	1.09 (0.71–1.68), *p* = 0.674
Sex ^1^	Female	Ref	Ref	Ref	Ref
	Male	1.05 (0.76–1.46), *p* = 0.748	1.15 (0.71–1.85), *p* = 0.566	0.96 (0.61–1.51), *p* = 0.874	0.96 (0.59–1.55), *p* = 0.871
Family history of CHD ^1^	No	Ref	Ref	Ref	Ref
	Yes	1.27 (0.91–1.77), *p* = 0.138	1.21 (0.55–2.68), *p* = 0.618	1.19 (0.82–1.74), *p* = 0.340	1.15 (0.78–1.70), *p* = 0.467
Arterial hypertension ^1^	No	Ref	Ref	Ref	Ref
	Yes	1.17 (0.88–1.56), *p* = 0.253	1.30 (0.77–2.19), *p* = 0.309	1.04 (0.73–1.48), *p* = 0.813	1.09 (0.75–1.57), *p* = 0.646
BMI ^2^	SD variation	0.15 (0.02–0.28), *p* = 0.018	0.15 (−0.05; 0.35), *p* = 0.142	0.13 (−0.03; 0.31), *p* = 0.123	0.16 (−0.01; 0.34), *p* = 0.075
DLCN score ^2^	SD variation	0.16 (0.03–0.29), *p* = 0.015	0.19 (0.00–0.39), *p* = 0.051	0.13 (−0.04; 0.30), *p* = 0.146	0.05 (−0.13; 0.23), *p* = 0.590
eLDLC ^2^	SD variation	0.20 (0.07–0.33), *p* = 0.002	0.25 (0.06–0.45), *p* = 0.011	0.18 (0.01–0.36), *p* = 0.036	0.14 (−0.04; 0.32), *p* = 0.133
eLDLC ^1^	≤190 mg/dL	Ref	Ref	Ref	Ref
	>190 mg/dL	1.36 (0.96–1.92), *p* = 0.070	1.98 (0.63–6.17), *p* = 0.187	1.20 (0.82–1.75), *p* = 0.339	1.09 (0.73–1.62), *p* = 0.667
HDLC ^2^	SD variation	−0.14 (−0.27; −0.01), *p* = 0.029	−0.06 (−0.26; 0.14), *p* = 0.540	−0.18 (−0.35; 0), *p* = 0.041	−0.21 (−0.39; −0.03), *p* = 0.023
Lipoprotein(a) ^2^	SD variation	0.05 (−0.08; 0.18), *p* = 0.463	−0.02 (−0.23; 0.17), *p* = 0.791	0.07 (−0.10; 0.25), *p* = 0.421	0.07 (−0.10; 0.26), *p* = 0.416

The association of the wPRS with the demographic and phenotypic characteristics was tested in each of the following groups: combined (PCHD + HC; n = 222), healthy control (HC; n = 97), premature coronary heart disease (PCHD; n = 125), and PCHD subgroup with non-definite clinical familial hypercholesterolemia (nFH, DLCN score ≤ 8; n = 117). The PCHD subgroup with definite clinical familial hypercholesterolemia (dFH, DLCN score > 8; n = 8) was not analyzed due to a small sample size. All results are expressed as a function of wPRS Z score (variation of the measured variable per 1 SD variation in the wPRS). ^1^ Results for binary variables are expressed as odds ratios obtained from logistic regression. ^2^ Before analysis, all continuous variables were standardized as Z scores and the results are expressed as standardized beta coefficients obtained from simple linear regression (SD variation of the measured variable per 1 SD variation in the wPRS). Ref—reference category.

**Table 4:** Univariate logistic model for odds of premature coronary heart disease (PCHD).

Variable	Measurement Level	Unadjusted OR (95% CI)	*p*-Value
Age	≤45 years	Ref	
	>45 years	2.75 (1.54–4.93)	0.0005
Sex	Female	Ref	
	Male	1.09 (0.56–2.12)	0.792
Family history of CHD	No	Ref	
	Yes	6.25 (2.78–14.07)	<0.0001
Arterial hypertension	No	Ref	
	Yes	2.95 (1.63–5.33)	0.0002
Body mass index	kg/m^2^	1.08 (1.01–1.15)	0.018
Body mass index ^1^	1st quartile	Ref	
	2nd quartile	0.84 (0.40–1.78)	0.655
	3rd quartile	1.68 (0.78–3.61)	0.181
	4th quartile	2.38 (1.08–5.21)	0.028
Smoking	No	Ref	
	Current/past	6.16 (3.42–11.07)	<0.0001
Alcohol consumption	No	Ref	
	Yes	0.53 (0.30–0.95)	0.034
DLCN score	Score unit	12.61 (6.05–26.28)	<0.0001
eLDLC	mg/dL	1.011 (1.006–1.017)	<0.0001
eLDLC	≤190 mg/dL	Ref	
	>190 mg/dL	8.23 (3.33–20.34)	<0.0001
HDLC	mg/dL	0.948 (0.927–0.970)	<0.0001
Lipoprotein(a)	mg/dL	1.016 (1.002–1.030)	0.016
Triglyceride	mg/dL	1.00 (0.99–1.01)	0.971
aPRS	SD	1.14 (0.87–1.49)	0.317
wPRS	SD	1.22 (0.93–1.59)	0.142

*p*-values were obtained by logistic regression analysis on the whole cohort (125 PCHD patients, 97 healthy control patients). ^1^ Body mass index quartiles (kg/m^2^): 1st quartile: 19.4–25.7; 2nd quartile: 25.7–28.7; 3rd quartile: 28.7–31.1; 4th quartile: 31.1–45.0. Abbreviations: aPRS—additive polygenic risk score; DLCN—Dutch Lipid Clinic Network; eLDLC—estimated low-density lipoprotein cholesterol level (before statin treatment); HDLC—high-density lipoprotein cholesterol; OR—odds ratio; wPRS—weighted polygenic risk score.

**Table 5:** Multivariate logistic models for odds of premature coronary heart disease (PCHD).

Variable	Measurement Level	Reduced Model		Complete Model	
		Odds Ratio (95% CI)	*p*-Value	Odds Ratio (95% CI)	*p*-Value
Alcohol consumption	No	Ref		Ref	
	Yes	0.25 (0.11–0.56)	0.0006	0.29 (0.13–0.67)	0.0036
Arterial hypertension	No	Ref		Ref	
	Yes	2.98 (1.44–6.17)	0.0032	2.42 (1.08–5.41)	0.0304
BMI	kg/m^2^	1.07 (0.98–1.16)	0.0938	-	-
Family history of CHD	No	Ref		Ref	
	Yes	10.35 (3.93–27.24)	<0.0001	10.01 (3.33–30.09)	<0.0001
Smoking (past/current)	No	Ref		Ref	
	Yes	10.09 (4.80–21.22)	<0.0001	10.86 (4.83–24.41)	<0.0001
eLDLC	mg/dL	-	-	1.012 (1.005–1.020)	0.0012
HDLC	mg/dL	-	-	0.949 (0.920–0.979)	0.0009
Lipoprotein(a)	mg/dL	-	-	1.020 (1.001–1.039)	0.0352
Model performance					
Overall model *p* value		<0.0001		<0.0001	
Cox and Snell R^2^		0.34		0.43	
AUC value (95% CI)		0.855 (0.802–0.899)		0.895 (0.847–0.932)	
Specificity		75.2%		78.3%	
Sensitivity		81.6%		84.8%	
Accuracy		78.8%		81.9%	

*p*-values were obtained by logistic regression analysis on the whole cohort (125 PCHD patients, 97 healthy control patients). Body mass index (BMI) was not excluded from the reduced model because it emerged as a marginally significant predictive factor: the extra sum-of-squares F test showed that the reduced model was marginally significantly better with than without BMI (AUCs 0.855 vs. 0.845, respectively; *p* = 0.09). However, BMI was excluded from the complete model due to its lack of significance (*p* = 0.48; AUCs with and without BMI 0.896 vs. 0.895, respectively).

**Table 6:** Multilayer perceptron neural network results for prediction of PCHD.

	Training Group		Testing Group		AUC			
	CC%	Comparison	CC%	Comparison	Median	IQR	Range	Comparison
Model 1	83.3	*p* = 0.02	82.6	*p* = 0.07	0.898	0.893–0.903	0.854–0.924	*p* = 0.34
Model 2	84.1		82.6		0.898	0.892–0.906	0.864–0.922	

The statistics generated here are based on 100 iterations of the multilayer perceptron neural network for each prediction model on the whole cohort (125 PCHD patients, 97 healthy control patients). For a comparison of CC% and AUC values, the Mann–Whitney test was used. Model 1 included the following variables: arterial hypertension, family history of CHD, smoking status, alcohol consumption, body mass index, HDLC, eLDLC, and Lp(a). Model 2: model 1 + wPRS. Abbreviations: AUC—area under the curve; CC—correct classification; (P)CHD—(premature) coronary heart disease; eLDLC—estimated pre-treatment LDLC; IQR—interquartile range; wPRS—weighted polygenic risk score.

## Data Availability

The data supporting the findings of this study are not publicly accessible due to sensitivity concerns but can be obtained from the corresponding author upon reasonable request.

## Supplementary Materials

The supporting information can be downloaded at: https://www.mdpi.com/article/10.3390/ijms251810038/s1.
