# Supplementary material for: An 8-SNP LDL Cholesterol Polygenic Score: Associations with Cardiovascular Risk Traits, Familial Hypercholesterolemia Phenotype, and Premature Coronary Heart Disease in Central Romania
Source: Int J Mol Sci. 2024 Sep 18;25(18):10038. doi: 10.3390/ijms251810038 (PMC11432653; doi:10.3390/ijms251810038)
Supplement: Supplementary file 1 [file ijms-25-10038-s001.zip › ijms-3162765-supplementary.pdf]

Supplementary Table S1. Allele frequency comparison between the studied Romanian population and other European populations

Reference SNP cluster ID	Gene	Minor allele	Common allele	Minor allele frequency						
				Current study (Romanian population)			Teslovich et al.[1] (Europeans)	Talmud et al.[2] (United Kingdom population)		
				HC (n=97)	PCHD (n=125)	All (n=222)	Meta-analysis ($n>10^5$)	FH - known mutation (n=319)	FH - no known mutation (n=321)	WHII controls (n=3020)
rs6511720	LDLR	T^{p}	G	0.118	0.064	0.087	0.110	0.100	0.080	0.130
rs629301	CELSR2	G^{p}	T	0.221	0.184	0.200	0.220	0.190	0.120	0.210
rs1367117	APOB	A*	G	0.345	0.304	0.322	0.300	0.350	0.370	0.330
rs4299376	ABCG8	G*	T	0.350	0.352	0.351	0.300	0.370	0.370	0.320
rs1800562	HFE	A^{p}	G	0.031	0.004	0.015	0.060	0.060	0.080	0.070
rs2479409	PCSK9	G*	A	0.350	0.368	0.360	0.300	0.330	0.390	0.350
rs429358	APOE	C	T	0.118	0.124	0.121	-	0.190	0.210	0.150
rs7412	APOE	T	C	0.072	0.048	0.058	-	0.040	0.030	0.080

*Risk (LDLC-raising) alleles. ^{p} Protective (LDLC-lowering) alleles. Abbreviations: FH – familial hypercholesterolemia HC – healthy controls, PCHD – premature coronary heart disease, SNP – single nucleotide polymorphism. References: [1]Teslovich et al. *Biological, Clinical, and Population Relevance of 95 Loci for Blood Lipids* (Nature, 2010) [4]; [2]Talmud et al. *Use of low-density lipoprotein cholesterol gene score to distinguish patients with polygenic and monogenic familial hypercholesterolaemia: a case-control study* (Lancet, 2013) [10].

Supplementary Table S2. Correlations between PRSs and the DLCN score and eLDLC levels.

	All (HC+PCHD) n=222	HC n=97	PCHD (nFH+dFH) n=125	PCHD nFH n=117	PCHD dFH* n=8
	**aPRS correlations**				
**With DLCN**	r_S = 0.120 p = 0.075	r_S = 0.223 p = 0.028	r_S = 0.086 p = 0.335	r_S = 0.043 p = 0.643	r_S = 0.304 p = 0.464
**With eLDLC**	r_S = 0.145 p = 0.030	r_S = 0.169 p = 0.098	r_S = 0.124 p = 0.167	r_S = 0.088 p = 0.342	r_S = -0.139 p = 0.743
	**wPRS correlations**				
**With DLCN**	r_S = 0.155 p = 0.020	r_S = 0.179 p = 0.079	r_S = 0.143 p = 0.111	r_S = 0.097 p = 0.298	r_S = 0.266 p = 0.524
**With eLDLC**	r_S = 0.205 p = 0.002	r_S = 0.195 p = 0.055	r_S = 0.187 p = 0.037	r_S = 0.149 p = 0.110	r_S = 0.024 p = 0.954
**With aPRS**	r_S = 0.858 p < 0.0001	r_S = 0.827 p < 0.0001	r_S = 0.883 p < 0.0001	r_S = 0.873 p < 0.0001	r_S = 0.962 p < 0.0001
	**aPRS vs wPRS (comparison of correlation coefficients)**				
**For DLCN**	p = 0.708	p = 0.753	p = 0.651	p = 0.682	p = 0.947
**For eLDLC**	p = 0.517	p = 0.853	p = 0.614	p = 0.640	p = 0.795

The two genetic risk scores (additive – aPRS, weighted – wPRS) were correlated with the Dutch Lipid Clinic Network (DLCN) scores and with the estimated LDLC levels (eLDLC, before statin treatment) across all participant groups: all (PCHD + HC), healthy controls (HC), patients with premature coronary heart disease (PCHD), patients with PCHD and no definite clinical familial hypercholesterolemia (nFH, DLCN score ≤8), and patients with PCHD and definite clinical FH (dFH, DLCN score >8). Due to the non-parametric distribution of data sets, the rank correlation test was used (r_s – Spearman's rho). *The results may be subject to unreliability due to the small sample size.

Supplementary Table S3A. Risk ratio for hypercholesterolemia (eLDLC >190 mg/dL) and definite clinical familial hypercholesterolemia (DLCN >8) for each aPRS decile.

aPRS decile	Mean aPRS (±SD, range)	Mean eLDLC (±SD, range)	Risk ratio (95% CI) of eLDLC >190 mg/dL	Median DLCN score (IQR), (range)	Risk ratio (95% CI) of DLCN >8
	**HC group (n=97)**				
Decile 1	4.90±0.31 (4-5)	114.0±25.8 (58.8-148.4)	Reference	0.00 (0.00-0.00) (0-1)	Reference
Decile 2	5.70±0.48 (5-6)	117.8±25.6 (86.8-149.1)	-	0.00 (0.00-0.00) (0-0)	-
Decile 3	6.66±0.50 (6-7)	132.8±38.5 (81.2-186.3)	-	0.00 (0.00-1.00) (0-2)	-
Decile 4	7.00±0.00 (7-7)	139.3±35.9 (88.9-218.9)	3.00 (0.13-65.9)	0.00 (0.00-1.00) (0-3)	-
Decile 5	7.00±0.00 (7-7)	140.9±24.2 (107.8-211.8)	3.00 (0.13-65.9)	0.00 (0.00-1.00) (0-4)	-
Decile 6	7.80±0.42 (7-8)	136.0±31.8 (90.8-196.9)	3.00 (0.13-65.9)	0.00 (0.00-1.00) (0-2)	-
Decile 7	8.00±0.00 (8-8)	125.3±38.8 (51.1-185.6)	-	0.00 (0.00-1.00) (0-1)	-
Decile 8	9.00±0.00 (9-9)	160.3±41.3 (106.5-250.9)	5.00 (0.27-92.6)	1.00 (0.00-2.00) (0-4)	-
Decile 9	9.00±0.00 (9-9)	127.0±34.0 (67.3-196.5)	3.30 (0.15-72.08)	0.00 (0.00-0.25) (0-3)	-
Decile 10	9.66±0.50 (9-10)	126.7±24.5 (87.3-165.2)	-	0.00 (0.00-1.00) (0-2)	-
	**PCHD group (n=125)**				
Decile 1	5.33±0.49 (5-6)	161.1±63.8 (66.1-285.2)	Reference	3.00 (2.00-5.00) (2-8)	Reference
Decile 2	6.00±0.00 (6-6)	124.7±43.6 (62.5-203.1)	0.61 (0.12-3.07)	3.00 (2.00-3.25) (2-6)	-
Decile 3	6.66±0.49 (6-7)	157.2±67.0 (60.8-309.8)	1.00 (0.25-3.99)	3.00 (2.50-5.00) (2-8)	-
Decile 4	7.00±0.00 (7-7)	212.6±93.2 (76.8-395.1)	2.46 (0.84-7.17)	5.00 (2.75-7.25) (2-10)	4.64 (0.24-87.91)
Decile 5	7.33±0.49 (7-8)	180.6±123.5 (63.1-523.0)	1.33 (0.37-4.72)	2.50 (2.00-6.00) (2-12)	3.00 (0.13-67.06)
Decile 6	8.00±0.00 (8-8)	168.5±118.1 (34.1-471.8)	1.23 (0.34-4.40)	3.00 (2.00-5.50) (2-11)	2.78 (0.12-62.48)
Decile 7	8.00±0.00 (8-8)	200.6±73.8 (106.5-376.5)	2.33 (0.78-6.94)	5.00 (2.00-6.50) (2-11)	3.00 (0.13-67.06)
Decile 8	8.53±0.51 (8-9)	182.5±79.6 (87.1-407.1)	1.23 (0.34-4.40)	3.00 (3.00-5.25) (2-10)	2.78 (0.12-62.48)
Decile 9	9.16±0.38 (9-10)	201.9±93.7 (93.1-345.2)	2.00 (0.64-6.20)	5.00 (3.00-8.00) (2-11)	5.00 (0.26-94.23)
Decile 10	10.23±0.43 (10-11)	152.0±46.0 (89.7-236.9)	0.92 (0.22-3.72)	3.00 (2.00-3.25) (2-5)	-

Supplementary Table S3A (continued).

aPRS decile	Mean aPRS (±SD, range)	Mean eLDLC (±SD, range)	Risk ratio (95% CI) of eLDLC >190 mg/dL	Median DLCN score (IQR), (range)	Risk ratio (95% CI) of DLCN >8
	**Combined group (HC + PCHD, n=222)**				
Decile 1	5.00±0.30 (4-6)	124.8±44.2 (58.8-285.2)	Reference	0.50 (0.00-2.00) (0-8)	Reference
Decile 2	6.00±0.00 (6-6)	142.4±61.7 (62.5-309.8)	4.00 (0.48-32.99)	2.50 (0.00-4.00) (0-8)	-
Decile 3	6.63±0.49 (6-7)	169.0±77.9 (76.8-395.1)	5.00 (0.63-39.39)	2.00 (0.00-4.00) (0-10)	5.00 (0.25-98.52)
Decile 4	7.00±0.00 (7-7)	146.9±54.3 (63.1-257.9)	6.00 (0.78-45.81)	2.00 (0.00-3.00) (0-7)	-
Decile 5	7.08±0.28 (7-8)	150.2±51.6 (60.8-297.1)	3.82 (0.46-31.62)	1.00 (0.00-2.75) (0-8)	-
Decile 6	8.00±0.00 (8-8)	174.1±119.0 (34.1-523.0)	6.00 (0.78-45.81)	2.50 (1.00-5.00) (0-12)	5.00 (0.25-98.52)
Decile 7	8.00±0.00 (8-8)	152.0±47.4 (83.3-252.7)	6.00 (0.78-45.81)	2.00 (1.00-3.00) (0-7)	-
Decile 8	8.68±0.47 (8-9)	155.5±72.8 (68.9-376.5)	4.00 (0.48-32.99)	3.00 (0.00-3.00) (0-11)	3.00 (0.12-69.76)
Decile 9	9.00±0.00 (9-9)	198.3±88.4 (67.3-407.1)	11.00 (1.55-78.09)	3.00 (0.00-6.00) (0-11)	7.00 (0.38-128.02)
Decile 10	10.04±0.47 (9-11)	144.1±39.9 (87.3-236.9)	2.87 (0.32-25.54)	2.00 (1.25-3.00) (0-5)	-

Supplementary Table S3B. Risk ratio for hypercholesterolemia (eLDLC >190 mg/dL) and definite clinical familial hypercholesterolemia (DLCN >8) for each wPRS decile.

wPRS decile	Mean wPRS (±SD, range)	Mean eLDLC (±SD, range)	Risk ratio (95% CI) of eLDLC >190 mg/dL	Median DLCN score (IQR), (range)	Risk ratio (95% CI) of DLCN >8
	**HC group (n=97)**				
Decile 1	0.349±0.149 (0.122-0.507)	116.7±25.6 (58.8-148.4)	Reference	0.00 (0.00-0.00) (0-1)	Reference
Decile 2	0.553±0.034 (0.511-0.601)	104.6±29.3 (51.1-154.9)	-	0.00 (0.00-0.00) (0-1)	-
Decile 3	0.666±0.048 (0.612-0.743)	125.6±28.0 (88.9-169.5)	-	0.00 (0.00-0.25) (0-1)	-
Decile 4	0.755±0.011 (0.743-0.773)	146.4±37.7 (87.1-211.8)	3.00 (0.13-65.90)	0.00 (0.00-1.00) (0-4)	-
Decile 5	0.800±0.015 (0.777-0.818)	131.4±22.9 (88.9-162.1)	-	0.00 (0.00-1.00) (0-2)	-
Decile 6	0.861±0.018 (0.832-0.885)	152.9±42.0 (108.5-218.9)	5.00 (0.27-92.6)	0.50 (0.00-1.00) (0-3)	-
Decile 7	0.904±0.013 (0.889-0.923)	133.8±21.14 (108.1-163.5)	-	0.00 (0.00-1.00) (0-2)	-
Decile 8	0.951±0.017 (0.925-0.975)	139.5±32.0 (83.3-196.9)	3.00 (0.13-65.90)	0.50 (0.00-1.00) (0-2)	-
Decile 9	0.988±0.010 (0.975-1.001)	137.1±56.1 (67.3-250.9)	5.50 (0.29-101.28)	0.00 (0.00-0.75) (0-4)	-
Decile 10	1.057±0.040 (1.013-1.118)	133.2±23.2 (87.3-165.2)	-	0.00 (0.00-1.00) (0-2)	-
	**PCHD group (n=125)**				
Decile 1	0.437±0.088 (0.291-0.526)	138.4±55.0 (60.8-237.5)	Reference	2.00 (2.00-4.00) (2-6)	Reference
Decile 2	0.612±0.038 (0.541-0.691)	146.8±75.3 (34.1-285.2)	1.23 (0.34-4.40)	4.00 (2.75-5.25) (2-8)	-
Decile 3	0.707±0.018 (0.691-0.743)	146.0±65.7 (76.8-309.8)	0.66 (0.13-3.30)	2.50 (2.00-5.00) (2-8)	-
Decile 4	0.774±0.018 (0.743-0.796)	169.6±49.9 (92.3-267.6)	1.53 (0.46-5.09)	3.00 (2.00-5.00) (2-8)	-
Decile 5	0.826±0.011 (0.814-0.848)	181.3±107.0 (63.1-395.1)	1.66 (0.50-5.46)	3.50 (2.00-6.00) (2-10)	5.00 (0.26-94.34)
Decile 6	0.875±0.016 (0.848-0.889)	208.9±112.4 (93.1-471.8)	1.53 (0.46-5.09)	4.00 (3.00-8.00) (2-11)	4.64 (0.24-87.91)
Decile 7	0.914±0.011 (0.889-0.923)	201.0±123.1 (68.9-523.0)	1.66 (0.50-5.46)	3.50 (2.00-7.00) (2-12)	3.00 (0.13-67.06)
Decile 8	0.961±0.019 (0.941-0.994)	177.1±59.8 (82.5-275.1)	2.15 (0.71-6.48)	5.00 (2.75-6.00) (2-8)	-
Decile 9	1.029±0.014 (0.994-1.047)	216.9±105.2 (89.7-407.1)	1.66 (0.50-5.46)	3.50 (3.00-9.00) (2-11)	7.00 (0.40-122.44)
Decile 10	1.117±0.049 (1.047-1.183)	154.4±43.4 (94.8-236.9)	0.92 (0.22-3.72)	3.00 (2.00-3.25) (2-5)	-

Supplementary Table S3B (continued).

wPRS decile	Mean wPRS (±SD, range)	Mean eLDLC (±SD, range)	Risk ratio (95% CI) of eLDLC >190 mg/dL	Median DLCN score (IQR), (range)	Risk ratio (95% CI) of DLCN >8
	**Combined group (HC + PCHD, n=222)**				
Decile 1	0.396±0.124 (0.122-0.522)	128.2±44.8 (58.8-237.5)	Reference	1.00 (0.00-2.00) (0-6)	Reference
Decile 2	0.581±0.037 (0.523-0.620)	122.5±57.2 (34.1-285.2)	1.00 (0.22-4.42)	1.50 (0-4) (0-8)	-
Decile 3	0.692±0.034 (0.622-0.743)	143.1±57.2 (76.8-309.9)	1.00 (0.22-4.42)	2.00 (1.00-3.00) (0-8)	-
Decile 4	0.763±0.015 (0.743-0.796)	156.2±41.9 (87.1-267.3)	1.33 (0.33-5.27)	1.50 (0.00-3.00) (0-8)	-
Decile 5	0.815±0.014 (0.796-0.837)	165.4±83.1 (63.1-395.1)	2.55 (0.77-8.39)	2.00 (1.00-3.75) (0-10)	4.79 (0.24-94.5)
Decile 6	0.869±0.017 (0.837-0.889)	170.7±84.3 (93.1-471.8)	1.66 (0.45-6.13)	2.50 (0.00-5.00) (0-11)	3.00 (0.12-69.87)
Decile 7	0.909±0.013 (0.889-0.923)	182.8±105.2 (68.9-523.0)	2.00 (0.57-7.00)	2.00 (1.00-7.00) (0-12)	5.00 (0.25-98.5)
Decile 8	0.954±0.016 (0.925-0.975)	154.0±44.9 (82.5-234.8)	2.33 (0.69-7.87)	2.00 (1.00-3.00) (0-6)	-
Decile 9	1.008±0.019 (0.975-1.038)	181.6±97.5 (67.3-407.1)	2.66 (0.81-8.74)	1.50 (0.00-6.00) (0-11)	7.00 (0.38-128.02)
Decile 10	1.095±0.048 (1.038-1.183)	151.8±37.5 (87.3-236.9)	0.95 (0.21-4.24)	3.00 (2.00-3.00) (0-5)	-

Note: The cohort (PCHD+HC) comprises 222 individuals, making it impossible to categorize them into 10 deciles with equal numbers of individuals. Additionally, individuals with the same PRS value may span across two consecutive deciles, particularly for aGRS, which has fewer possible values than wGRS (refer to the explanatory note* in Supplementary table 5). In the analysis presented here, we aimed to delineate the categories as accurately as possible while maintaining a balanced number of individuals in each decile. In contrast, Supplementary table 9 prioritizes the separation of individuals based on wPRS over maintaining equally populated deciles. This approach ensures that all individuals with the same wPRS are grouped into a single decile, even if they span two consecutive deciles, resulting in an uneven numerical distribution across deciles. Consequently, the wPRS value ranges for each decile in the combined group (PCHD+HC) in this analysis are highly similar, but do not always match those in Supplementary Table 9. The upper limits of the wPRS for each decile here, compared to Supplementary Table 9, are as follows: decile 1 (both 0.522), decile 2 (0.620 vs 0.622), decile 3 (both 0.743), decile 4 (0.796 vs 0.780), decile 5 (both 0.837), decile 6 (both 0.889), decile 7 (0.923 vs 0.925), decile 8 (both 0.975), decile 9 (1.038 vs 1.030), decile 10 (both 1.183).

Supplementary Table S4. Trends in eLDLC level and DLCN score variation with PRS deciles

	All (HC+PCHD)* n=222	HC n=97	PCHD (nFH+dFH) n=125	PCHD nFH n=117	PCHD dFH n=8
	**eLDLC level trend**				
**As per aPRS deciles**	p = 0.031	p = 0.165	p = 0.192	p = 0.637	-
**As per wPRS deciles**	p = 0.0019	p = 0.046	p = 0.036	p = 0.076	-
	**DLCN score trend**				
**As per aPRS deciles**	p = 0.099	p = 0.148	p = 0.396	p = 0.994	-
**As per wPRS deciles**	p = 0.037	p = 0.121	p = 0.138	p = 0.277	-

To further examine the relationship between polygenic scores and phenotypic aspects (DLCN score and eLDLC level), we segmented the data into polygenic risk score (PRS) deciles. Then, we employed the Jonckheere-Terpstra test to explore potential trends across various groups: the combined group (all participants: HC+PCHD), healthy controls (HC), patients with premature coronary heart disease (PCHD), and patients with PCHD and no definite clinical familial hypercholesterolemia (nFH, DLCN score ≤8). Data for PCHD patients with definite clinical FH (dFH, DLCN score >8) could not be divided into deciles due to a limited sample size.

*Before conducting the Jonckheere-Terpstra test, which is sensitive to data ordering, the dataset was organized in two hierarchical levels. Initially, the sorting was done based on the PRS, from the smallest to the largest values. This sorting was necessary for establishing PRS deciles. Subsequently, within groups sharing the same PRS value, the data was shuffled using a randomizing function. This randomization was particularly important for the aPRS, where the limited range of values (aPRS range: 4-12, integers only) caused certain more frequent aPRS values to span across multiple deciles. Furthermore, considering the varied impact of each randomization on statistical significance, we conducted 10 cycles of randomization and statistical analysis. The resulting average p value is displayed in the table, while the individual p values from each randomization are provided here: aPRS/ eLDLC (all below 0.05: 0.027, 0.040, 0.013, 0.033, 0.047, 0.044, 0.039, 0.014, 0.039, 0.019), aPRS/ DLCN (all above 0.05: 0.072, 0.075, 0.086, 0.107, 0.109, 0.109, 0.095, 0.129, 0.112, 0.095), wPRS/ eLDLC (all below 0.05: 0.00220, 0.00250, 0.00148, 0.00130, 0.00260, 0.00273, 0.00122, 0.00105, 0.00281, 0.00142), and wPRS/ DLCN (all below 0.05: 0.041, 0.049, 0.025, 0.035, 0.040, 0.045, 0.033, 0.032, 0.037, 0.038).

Supplementary Table S5. Results of principal component analysis

	Rotated component matrix		Rotations sums of squared loadings		
			**Total**	**% of variance**	**Cumulative %**
**Component 1**	HDLC	-0.768	1.957	17.793	17.793
	Body mass index	0.729			
	Triglyceride	0.645			
	Arterial hypertension	0.521			
**Component 2**	eLDLC	0.742	1.297	11.787	29.580
	wPRS	0.666			
**Component 3**	Sex	0.806	1.274	11.581	41.161
**Component 4**	Smoking	0.682	1.254	11.402	52.563
	Family history of CHD	0.643			
	Alcohol consumption	0.484			
**Component 5**	Lipoprotein(a)	0.827	1.136	10.328	62.891

Subgroup: PCHD (n=125).

Kaiser-Meyer-Olkin measure of sampling adequacy: 0.558.

Bartlett's test of sphericity: < 0.001.

Extraction method: principal component analysis.

Rotation method: Varimax with Kaiser normalization.

Rotation converged in 8 iterations.

Supplementary Table S6. Odds ratio for PCHD of those above the 90th wPRS decile (n=24, wPRS ≥1.038)

Reference group	n	wPRS range	OR (95% CI)	p-value
**Within the 10th percentile**	22	≤ 0.522	3.60 (1.03-12.54)	0.042
**Within the 20th percentile**	45	≤ 0.622	3.13 (1.05-9.36)	0.037
**Within the 30th percentile**	71	≤ 0.743	2.46 (0.87-6.93)	0.084
**Within the 40th percentile**	87	≤ 0.780	2.67 (0.96-7.38)	0.053
**Within the 50th percentile**	112	≤ 0.837	2.50 (0.92-6.79)	0.065
**Within the 60th percentile**	137	≤ 0.889	2.40 (0.90-6.43)	0.074
**Within the 70th percentile**	156	≤ 0.925	2.38 (0.89-6.31)	0.076
**Within the 80th percentile**	178	≤ 0.975	2.45 (0.92-6.46)	0.064
**Within the 90th percentile**	198	≤ 1.030	2.55 (0.97-6.69)	0.051

Note: The wPRS value ranges for each decile reported here are highly similar but do not always match those in Supplementary Table 4B for the combined group (PCHD+HC). The upper limits of the wPRS for each decile here, compared to Supplementary Table 4B, are as follows: decile 1 (both 0.522), decile 2 (0.622 vs 0.620), decile 3 (both 0.743), decile 4 (0.780 vs 0.796), decile 5 (both 0.837), decile 6 (both 0.889), decile 7 (0.925 vs 0.923), decile 8 (both 0.975), decile 9 (1.030 vs 1.038), decile 10 (both 1.183). For further explanation, refer to the explanatory note below Supplementary Table 4B.
